# Supplementary material for: Are carbon emissions trading and green financial instruments synergistic? -Comprehensive quantitative research based on content analysis
Source: PLoS One. 2024 Mar 7;19(3):e0298601. doi: 10.1371/journal.pone.0298601 (PMC10919601; doi:10.1371/journal.pone.0298601)
Supplement: S1 Data — (ZIP) [file pone.0298601.s001.zip › Data/地级市Z_F工作报告.zip!/╣≤╓▌/┴∙┼╠╦«╩╨/└┤╫╘╣1⁄2╓┌║┼í╛┬φ┐╦╩2╛▌═°í┐_2009.docx]

各位代表：

　　现在，我代表市人民政府，向大会作工作报告，请予审议，并请各位政协委员和列席的同志提出意见。

　　２００８年政府工作回顾

　　过去一年，是我市发展进程中很不寻常的一年。面对百年不遇的特大雪凝灾害、历史罕见的国际金融危机等重大挑战和考验，我们坚持以邓小平理论、“三个代表”重要思想为指导，深入贯彻落实科学发展观，在省委、省政府和市委的坚强领导下，在市人大、市政协的监督、支持和帮助下，带领全市各族人民，群策群力，排难而进，扎实工作，全市经济社会发展呈现出增长较快、结构优化、效益提高、物价回落、民生改善的良好态势。

　　第一，经济保持平稳较快发展。全市生产总值完成３８４．２７亿元，增长１４．９％，人均生产总值首次突破万元大关，达到１２５１６元。全社会固定资产投资完成１９７．７亿元，增长１６．２％。财政总收入完成６１．４１亿元，其中地方财政收入完成２９．７１亿元，分别增长３３．２８％和３７．５２％。社会消费品零售总额完成９３．８１亿元，增长２０．９％。年末金融机构存、贷款余额分别为３４１．８９亿元和２２３．９９亿元，分别比年初增加７１．４４亿元和１７．３６亿元。

　　第二，农业农村经济加快发展。粮食产量达到８４．１６万吨，增长２．４％。以马铃薯、蔬菜、畜牧养殖“三大产业”为重点的农业结构调整取得新进展，规模化水平和商品率进一步提高，马铃薯种植面积达１５１．５万亩，新增商品蔬菜基地３．０７万亩，畜牧业产值在农业总产值中的比重较上年提高１．０８个百分点。“三农”投入加大，各项强农惠农政策得到较好落实，城市、企业、民间的资金、技术、人才加速向农村流动，农业社会化服务体系加快建立和完善，村庄整治取得新成效。新阶段扶贫开发继续推进，解决了３．９万农村贫困人口的温饱问题。大力开展“阳光工程”、“绿色证书”、“雨露计划”等各类农业适用技能培训，农民致富的愿望和能力得到增强。全年农民人均纯收入达到２７３４．９４元，增长１６．５％，剔除价格上涨因素，实际增长７．６％。

　　第三，工业经济增长的质量和效益提高。工业增加值完成２２６亿元，其中规模以上工业增加值完成１７５．３９亿元，分别增长２０．６％和２４．５％，工业经济对全市经济发展的贡献率达７４．７％。煤、电、钢、建材等支柱产业进一步做大做强，煤炭产量、发电量和钢材产量分别增长６％、３２．６％和４．３％，新增新型水泥干法生产能力１８０万吨。煤化工、矿山机电装备制造业、有色金属工业、轻工业等加快发展，产业门类和产品结构日益优化合理，产品附加值不断提高。新型工业化步伐加快，更加注重节能减排，增长方式加快由粗放向集约转变，万元工业增加值能耗和单位ＧＤＰ能耗分别完成省下达的任务。预计二氧化硫排放量和化学需氧量排放量分别较上年下降１１．４７％和５．４９％。

　　第四，基础设施建设迈出重要步伐。贵昆客运专线纳入国家中长期铁路网规划，六沾铁路复线建设顺利。水盘高速公路及在建二级公路建设加快，建成通乡油路２００公里和通村公路７６０公里。六盘水月照机场已进入开工建设前的准备阶段。水利建设取得新进展，解决了农村１１．４万人和１．８１万头牲畜的饮水安全问题；黔中水利枢纽工程、白河沟供水工程获准建设。编制了全市电网三年建设规划，一批输变电工程建成投入使用。城市路网、水电气和通信管网、绿化美化、文化体育、垃圾和污水处理等设施建设力度加大，城市功能进一步完善。石漠化综合治理项目启动实施。林业生态建设扎实推进，森林覆盖率达到３６．０２％。紧紧抓住中央扩大内需促进经济增长的有利时机，已争取到第一批中央直接投资２．８４亿元，一批重大基础设施项目相继开工建设。

　　第五，改革开放继续深入推进。国有企业改革改制稳步开展，市水务公司启动组建。农村综合改革继续深化，农村集体林权制度改革有序进行。投融资体制改革步伐加快，六盘水市商业银行挂牌，邮政储蓄银行六盘水分行成立，小额贷款公司组建工作顺利。非公有制经济发展加快，实现增加值和税金分别增长１３％和１８％。隆重举办了纪念改革开放暨建市３０周年庆典活动。与对口帮扶和友好城市、周边地区的交往日益加深。招商引资规模不断扩大，全年招商引资实际到位资金８０亿元，增长２０％；外贸进出口总额累计完成４．２５亿美元，增长４．４％。沃尔玛、肯德基、国贸等国内外知名连锁企业相继入驻。

　　第六，民生问题得到较好保障和改善。新增城镇就业１．９６万人，城镇登记失业率控制在４．３％以内。城镇居民人均可支配收入达到１２１４２．１９元，增长９．１％，剔除价格上涨因素，实际增长４％。城镇居民基本医疗保险全面启动实施。新型农村合作医疗覆盖面进一步扩大，参合率达９４．２％。基本养老、基本医疗、失业、工伤、生育保险的扩面征缴任务超额完成。城市低保月人均补助水平增加３４元，农村低保年人均补助标准从３６０元提高到４６０元，城乡低保实现应保尽保、分类施保。国有重点煤矿采煤沉陷区综合治理工程有力推进；８０００户农村茅草屋改造和１２００户农村危房改造全面完成；人均住房建筑面积不足１０平方米的城市低收入家庭基本实现应保尽保。启动实施临时价格干预措施，抑制了物价大幅波动。安全隐患排查治理深入开展，安全生产形势持续稳定好转，全市煤矿百万吨死亡率、道路交通万车死亡率、亿元ＧＤＰ生产安全事故死亡率和工矿商贸企业就业人员１０万人生产安全事故死亡率分别较上年下降０．１９、１．５８、０．３和１．４５。强化食品药品质量安全监管，有效应对和处置了食品药品安全突发事件。社会治安、信访、应急管理等进一步加强，“五五”普法力度加大，毒品重点整治取得新成效，创建“平安六盘水”工程稳步推进，全市社会保持稳定。

　　第七，基本公共服务均等化水平稳步提高。各类教育加快发展，六盘水师专“升本”工作进展顺利；“两基”迎“国检”工作扎实推进；城乡义务教育阶段学杂费全部免除；共发放农村义务教育阶段家庭经济困难寄宿生生活费补助２８８３万元。地氟病防治工作成效明显，完成防氟改灶任务７万户；建成３１２个村卫生室；对２５０名乡村医生进行了中专学历培训，对１０９５名村卫生员进行了中医理论和实践知识培训。公共文化服务体系建设加快，一批县乡文化基础设施启动建设，文物和非物质文化遗产保护力度加大；完成了市中心城区５万户数字电视整体转换平移工作和全市农村广播电视节目无线覆盖工程。人口计生工作水平稳步提高，人口出生率为１２．７‰，符合政策生育率达９５．６％。组团参加贵州省首届农民运动会，取得了奖牌数第一的优异成绩。老龄、妇幼和残疾人事业取得新进步。人事人才工作取得新进展。科技、民族、宗教、外事、对台、档案、地方志、气象、人防、新闻出版等工作取得新成绩。

　　第八，民主法制建设和政府自身建设进一步加强。自觉接受人大的法律监督、工作监督和政协的民主监督。高度重视民主党派、工商联和无党派人士的意见和建议，注重发挥工会、共青团、妇联等人民团体的参与监督作用。大力推行重大事项决策公示和听证制度，通过多种形式主动征求专家学者和人民群众的建议，民主决策水平不断提高。认真贯彻《政务信息公开条例》，政务公开、厂务公开、村务公开和部门办事公开继续扩大。新闻发布制度逐步完善。电子政务建设不断加强。行政审批制度改革稳步推进。出台了《六盘水市人民政府工作规则》、《六盘水市人民政府部门行政首长问责试行办法》，行政执法行为进一步规范。审计、监察、廉政建设和反腐败工作力度加大，政风行风进一步好转。大力弘扬“一方有难、八方支援”的优良传统，积极参与和支援了四川汶川等地震灾区的抢险救灾。

　　各位代表！一年来，我们经受住了严峻的考验，积累了应对复杂形势的宝贵经验。取得抗凝救灾重大胜利的实践证明，只要我们万众一心、众志成城，就一定能够排除万难、共克时艰；积极有效应对国际金融危机带来不利影响的实践证明，只要我们时刻与党中央保持高度一致，认清形势，坚定信心，统筹兼顾，未雨绸缪，就一定能够化挑战为机遇、变压力为动力，推动全市经济平稳较快发展；一批盼望已久的重大项目相继开工建设的实践证明，只要我们敢于破除传统的思维定式，以只争朝夕、时不我待的昂扬斗志，集中精力谋大事、创大业，创造性地开展工作，就一定能够破解发展难题，把我市改革开放事业不断推向前进。

　　各位代表！过去一年取得的成绩来之不易，这是省委、省政府坚强领导，省直各部门大力支持的结果；是全市各族干部群众共同奋斗，社会各方关心支持的结果。在此，我代表市人民政府，向全市工人、农民、知识分子、干部，向各民主党派、工商联、无党派人士、人民团体，向驻市人民解放军指战员、武警部队官兵、公安政法干警，向所有关心和支持我市改革发展的各界人士，表示衷心的感谢并致以崇高的敬意！

　　在看到成绩的同时，我们也清醒地认识到，经济社会发展中的一些主要矛盾和问题依然存在：农民持续稳定增收渠道不多，城乡二元结构突出，统筹城乡发展难度较大；企业改革改制脱困任务较重；粗放的经济发展方式还未从根本上得到改变，节能减排与科学发展的要求还有差距；交通、水利、电网等基础设施建设滞后于经济社会发展；就业、就学、就医、社会保障等民生问题还未得到妥善解决；部分企业安全工作基础薄弱，生产安全事故时有发生，道路交通事故多发状况尚未改变；矿群矛盾、工农纠纷、征地拆迁、涉法涉诉、社会治安等问题较为突出，维护社会稳定压力较大。对于这些问题，我们将采取有力措施认真加以解决。

　　２００９年政府工作的主要任务

　　今年是新中国成立６０周年，也是推进“十一五”规划顺利实施的关键一年。政府工作的总体要求是：全面贯彻党的十七大、十七届三中全会精神，以邓小平理论和“三个代表”重要思想为指导，深入贯彻落实科学发展观，进一步解放思想，抢抓机遇，以扩大内需、保持经济平稳较快发展为首要任务，加速工业化、加快城镇化、推进农业现代化，着力扩大投资规模，调整经济结构，转变发展方式，深化改革开放，保障改善民生，加强社会建设，推动全市经济社会又好又快发展。

　　全市经济社会发展的主要预期目标是：生产总值增长１３％以上；全社会固定资产投资增长２０％以上；财政总收入和地方财政收入增长１８％以上；社会消费品零售总额增长１７％以上；城镇居民人均可支配收入增长８％以上，农民人均纯收入实际增长８％以上；居民消费价格总水平涨幅控制在４％以内；城镇登记失业率控制在４．５％以内；人口出生率控制在１２．８‰以内；单位生产总值能耗下降４．７１％，主要污染物减排２％。

　　实现以上目标，虽然面临国际金融危机及其影响的严峻考验和挑战，但总的来看，依然是希望多于困难、机遇大于挑战。中央进一步扩大内需促进经济平稳较快发展的十条措施，为我市加快发展提供了强有力的政策、项目、资金等保障；扩大内需将加大对我市资源性产品的需求；受宏观政策调整和成本因素推动，产业、企业整合成本下降，有利于推动产业转型升级，促进发展方式转变，加速资源优势向经济优势转化；改革开放３０年的持续快速发展，我市形成了较为稳固的产业基础，具备了一定抵御风险的能力。因此，只要我们辩证地认识面临的形势，防止盲目乐观，增强忧患意识，坚定发展信心，积极主动应对，就能够危中寻机，转危为机，实现更好更快发展。

　　今年，我们将围绕“抓投资，调结构，促生产，上消费，保民生，维稳定”，着力抓好以下九个方面的工作。

　　（一）千方百计扩大投资规模，充分发挥投资对扩大内需的拉动作用。抢抓中央增加投资机遇，以“政府投一块、市场融一块、借地生一块、银行贷一块、向上争一块”为工作方向，加快形成投资主体多元化格局，保持相当的投资规模。

　　按照“出手要快，出拳要重，措施要准，工作要实”的要求，以项目为载体，大抓项目、抓大项目，力争得到国家和省的更多投入，促进大发展。创新机制，坚持“急事急办，特事特办”的原则，实行“一站式”、“一条龙”等工作方式，抓紧完善项目前期工作，争取上半年申报更多规模大、投资多、带动力强的项目。着力在缓解土地和融资的制约方面取得突破性进展，促成项目早日落地建设。加强项目建设的协调服务，建立完善一个项目、一名领导、一套班子、一个方案、一抓到底的推进机制，确保项目顺利建设，形成更多的实物工程量。

　　在努力争取中央和省加大投入的同时，多渠道筹集资金，尤其是在建立健全还贷机制的前提下，适度负债加大投入，启动实施一批我市自身发展需要的建设项目。一是调整财政支出结构。既保证中央和省下达项目的必要配套资金，又整合各类投入，坚持勤俭办一切事业，努力减少不必要的开支，集中财力办大事、办实事。二是拓宽融资渠道，在创新融资平台、推出融资项目等方面见到实效。大力发展资本市场，鼓励和支持符合条件的企业上市，努力在地方政府发债工作、地方金融机构推出理财产品等方面有所突破。加强政银合作，推动与中国建设银行贵州省分行１５０亿元贷款合作框架协议顺利落实。三是激活社会投资，通过以资源换项目、以项目换资金、以产权换资金、以存量换增量、以市场换资金等多种形式，引导、鼓励、支持民间资本对政府鼓励的项目和符合国家产业政策的领域加大投入。四是以增值税转型改革为契机，鼓励企业加大技改投入，提升企业综合竞争力。

　　加强项目的储备和管理。把项目储备作为保证投资持续增长的基础工作来抓，加大项目前期费投入，适时储备一批对经济社会发展有重大影响和带动作用的项目。实行严格的项目管理制度，开展项目清理，对有项目无资金、借项目圈土地占资源等情况进行清查；建立健全重大项目安全保障体系和廉政保障体系，全面推行和不断完善项目法人责任制、招投标制度、工程监理制度和合同管理制度，提高项目建设管理的程序化、规范化、科学化水平。加强资金监管，严防将投资用于不符合规定投向、不符合管理要求的项目建设。

　　在统筹抓好各方面投资的同时，突出抓好交通、水利、电网等基础设施建设，尽快改善发展条件。交通方面，以５年实现各县、特区、区通高速公路为目标，加快水盘高速公路建设，抓紧做好大山哨经水城至昭通高速公路前期工作并争取大山哨至六枝段开工建设，扎实推进杭瑞高速公路境内段、“毕水兴”高速公路盘县至兴义段、纳雍经六枝至晴隆等高速公路前期工作；建成红威、英柏、柏火等二级公路，推进玉马公路建设，开工建设红果新城环城东线、姚家营至大湾、英武至大山、英武至羊场等二级公路；加快建设大河至木果、陡箐至化乐、响水河至三块田等运煤公路，新建新华至大草坝、长箐经黑塘至新场、中寨至捞河、猴场经果布嘎和野钟至杨梅、化乐经比德至立火、新民至楼下等一批运煤公路；建设天生桥旅游公路；继续建好通乡油路和通村公路；配合做好六沾铁路复线、贵昆客运专线建设的协调服务；启动市中心城区城市轻轨项目前期工作；加快建设六盘水月照机场；开工建设野钟、中寨码头。水利方面，积极主动做好黔中水利枢纽一期工程建设的有关工作，加快建设白河沟供水工程，争取双桥供水工程、六盘水大型灌区节水改造工程开工建设；完成病险水库治理任务；实施好饮水安全工程，解决农村１１万人饮水安全问题；建设７．７万亩烟水配套工程；结合石漠化治理，继续抓好水土保持治理。电网方面，提前实施“十二五”电网规划；着力完善地方煤矿双回路供电设施；开工建设１个５００ＫＶ输变电工程和３个２２０ＫＶ输变电工程，建成５个１１０ＫＶ输变电工程；加强农村电网建设和改造，力争通电率达１００％。

　　（二）以农民增收为核心，加快发展农业农村经济。全面贯彻落实中央１号文件精神，坚持“工业反哺农业、城市支持农村”和“多予少取放活”的方针，充分发挥农民的主体作用，按照“二十字方针”的要求，加快建设社会主义新农村。

　　以发展现代农业为基本方向，大力调整农业结构，推动农业产业化，不断提高农业综合生产能力。一是突出大种植，发展优势特色产品。毫不放松粮食生产，稳定播种面积，提高复种指数，着力在推广良种良法、优化品种结构、提高单产上下功夫，力争粮食产量增产２％以上。进一步扩大马铃薯种植面积，力争马铃薯种植面积达１８０万亩以上。以城郊商品蔬菜基地建设为重点，新增商品蔬菜基地２万亩以上，并根据市场需求积极发展反季节蔬菜、无公害蔬菜。因地制宜发展烤烟、茶叶、水果、中药材等经济作物，加快形成新的农村区域性支柱产业。二是突出大养殖，确保畜牧业产值在农业总产值中的比重提高１个百分点以上。抓好优质肉牛、肉羊、肉猪、肉禽基地建设和畜禽良种推广，认真兑现畜牧养殖补贴和扩大保险覆盖面，加强动物防疫工作，增强畜牧业抗风险能力；抓好草畜配套、圈舍配套，完成人工牧草种植５万亩；新建牛改点２６个；规划启动一批规模养殖场和养殖小区，提高畜牧业发展的规模化、集约化、专业化水平。三是突出大加工，培育壮大龙头企业，重点发展粮食、畜产品、马铃薯、生姜、辣椒、茶叶等农产品的精深加工。加大对企业的扶持力度，通过招商引资建设上规模、上档次、科技含量高、辐射带动力强的大型农产品加工企业，力争更多的企业进入省级、市级重点龙头企业行列。四是突出大市场，提高农副产品商品率。以建立紧密相连、城乡互动的市场体系为目标，畅通农产品运输“绿色通道”，消除城乡市场分割，促进物资双向流动。加强农业社会化服务体系建设，充分发挥农民经纪人队伍和各类农业专业协会在服务生产、搞活流通、开拓市场等方面的重要作用。整合农村信息资源，发展农产品现代流通方式，加快建设产地农产品批发市场，为农产品进入市场创造便捷条件。

　　加大对农业的支持保护力度。认真落实各级党委政府的强农惠农政策，保证各项涉农补贴一分不少地及时发放到农民手中。继续加大对“三农”的投入，强化资金监管，保证各项支农资金一分不少地用在发展农业农村经济上。加大农业综合开发力度，实施好１．８万亩中低产田土改造和３０００亩坡改梯工程，建设１３３４０亩退耕还林基本口粮田，建成沼气池２万口。推动金融服务机构加快向农村延伸，早日实现每个乡镇都有金融网点。继续开展“阳光工程”、“绿色证书”、“雨露计划”等培训，完成农业适用技术普及培训４２万人（次）以上。制定相关激励政策，鼓励和引导各方面的资金、技术、人才参与新农村建设，形成全社会关心支持“三农”工作的良好氛围。

　　深入推进新阶段扶贫开发。坚持先易后难、样板先行，按照打捆使用、滚动发展的资金使用原则，以基础设施建设、培育特色优势产业和提高人口素质为重点，大力实施开发式扶贫，不断增强贫困地区自我发展能力，全面完成减少农村贫困人口３．８３万人的任务。

　　（三）大力调整经济结构，推动资源优势向经济优势转化。把结构调整作为保增长的主攻方向，丰富产业门类和产品种类，促进发展方式转变，增强发展后劲。

　　第一，优化产业结构。在巩固和加强农业的同时，努力在以下方面取得新进展。一是在做大做强支柱产业方面取得新进展。以促使煤矿上规模、上档次、上水平为目标，调整煤矿结构，促进煤炭企业重组，深入开展大矿帮扶小矿，建成一批大中型骨干矿井，扎实推进玛依、化乐等大中型煤矿的前期工作，使全市煤炭生产能力达５５００万吨以上；全面建成发耳电厂，开工建设坡上牧场风力发电项目，力争开工建设六枝电厂、盘南电厂三期工程及一批水电项目，新增电力装机容量１２０万千瓦以上；支持首钢水城钢铁（集团）有限责任公司扩能改造，开工建设贵州盘县“煤（焦、化）－钢－电”一体化循环经济工业基地项目；建成盘县三合水泥有限责任公司等一批水泥项目，使水泥生产能力达到５００万吨以上。二是在新兴支柱产业的培育和发展方面取得新进展。加快发展煤化工，建成老鹰山煤基气化替代燃料项目一期工程，兴建盘县红果循环经济型煤焦化等一批煤化工项目；以建成贵州矿山机械和机电装备制造业基地为目标，支持水城矿业（集团）公司机械制造分公司、盘江装备制造公司、贵州宏狮煤机制造有限公司等企业加快发展，务求在产品开发、生产和销售上取得新成效。三是在服务业发展方面取得新进展。继续大力发展现代金融、保险、物流以及教育培训、医疗、卫生等成长潜力大，并已有良好基础的重点服务业。加快旅游业的发展，围绕建设六盘水乌蒙山国家地质公园，着力在北盘江大峡谷上做文章；研究保护利用好珍稀动物黑叶猴；推进牂牁湖景区建设；以满足城市居民消费需求为出发点，开发以城郊农家乐为主的乡村旅游，尽快在城郊形成农家乐旅游带和产业带；以繁荣农村消费为目的，组织好“家电下乡”，加快“万村千乡市场”和“双百市场”工程建设，不断完善农村消费设施和服务体系。

　　第二，优化产业布局。立足资源、交通等条件，合理调整和布局各类产业，形成产业集群和产业带，以大产业促进大发展。切实把工业集中区作为优化产业布局的关键，把握世界范围内的“第五次产业转移”机遇，抓紧在各县、特区、区分别规划建设一个工业集中区，推动轻工业和装备制造业向园区集聚，尽快把工业集中区打造成为承接产业转移的载体，成为产业聚集、城市发展的新组团。今年重点推进红山、石桥工业集中区建设，动工建设红山石桥一号主干道，同步建设电力、通讯、给排水等基础设施，为企业入驻创造条件。

　　第三，优化组织结构。重视和发挥大企业和非公有制企业在经济结构调整中的主体作用。搞好协调服务，积极为大企业发展创造良好环境。鼓励和支持企业之间开展强强联合，促进企业投资主体和产权多元化，重点促成煤炭、电力、钢铁、化工等企业形成战略合作伙伴，组建大型企业集团，形成“大煤保大电、大煤保大钢、大煤保大化”的有利格局。继续从融资、管理、培训、项目、信息等方面加强对非公有制企业的引导、支持和服务，鼓励非公有制企业通过兼并、合作等方式实现规模化、多元化发展，尽快建立现代企业制度。大力实施中小企业成长工程，积极为企业排忧解难。

　　（四）加快城镇化步伐，推进城乡一体化进程。以形成大中城市和小城镇协调发展的城镇体系为目标，围绕打造交通便捷、环境优美、教育卫生等服务功能完善的四季宜居城市，按照规划、建设、管理“三位一体”的要求，突出扩容和宜居两个重点，加大市中心城区和平寨、红果两个次中心城市建设力度，有序推进小城镇建设。

　　着力抓好城镇规划。在《六盘水城市总体规划（２００６－２０２０）》的指导下，抓紧编制各级各层次城乡规划。加强市中心城区“一城七片”控制性规划和重要地段、重要节点、重要建筑修建性详规的编制，突出编制好水月片区、德坞片区、松坪凤凰组团及旧城改造的规划。扎实抓好盘县和六枝特区的城市总体规划修编。有序推进小城镇规划修编，配合贵州盘县“煤（焦、化）－钢－电”一体化循环经济工业基地项目建设，启动盘县鸡场坪小城镇建设规划，协调推进重大项目与重点小城镇建设。

　　着力抓好城市扩容。以城市道路建设为扩容的先行举措，通过路网建设拉开城市骨架，拓展城市发展空间。编制完成并启动实施市中心城区路网规划，改造火车站并完善以北片区路网。在继续建好东西向主干道的同时，把重点转移到南北向主干道建设上来，并统筹其他道路建设，配套完善水、电、气、通讯等基础设施。全面建成凉都大道西段，基本建成人民路东段，完成机场路一期工程基础部分；开工建设钢城大道北段，启动汪水路改造工程；推进市中心城区入城口改造；开展人民路西段、向阳北路延伸段、车站路、官厅路建设的前期工作并尽快启动建设；实施小街小巷综合改造三期工程。

　　着力抓好城市人口聚集。把机械性人口增长作为提高城镇化水平的核心措施来抓。一是通过加强保障性住房工程建设“安置人”。加大廉租房、经济适用房、农民工公寓等建设力度，解决低收入家庭和进城务工人员的住房问题；有序推进“城中村”和旧城改造。二是通过完善城市功能“吸引人”。建成市三中凤凰校区、市一中双水校区、市人民医院住院大楼主体工程等项目；加快凉都体育中心建设步伐；高起点编制凤凰山文化公园规划，新建市图书馆、市博物馆；建成２２个公厕、１４个垃圾中转站和市中心城区民用管道煤气第二气源厂输配工程；开展城市集中供暖的前期工作；采取市场化方式，尽快建成一批停车场，缓解停车难和交通无序的状况。三是通过深化户籍制度改革“转移人”。深入宣传户籍制度改革政策，简化落户程序和手续，促进在城镇有稳定职业和住所的进城务工人员、因征地失去生活保障的农村人口向城镇流动。四是通过繁荣城市经济、扩大就业“稳定人”。

　　着力加快小城镇建设。在小城镇规划的指导下，有序实施工矿区的城镇化改造。鼓励和扶持村镇建设，将小城镇建设与煤矿棚户区改造、国有重点煤矿采煤沉陷区综合治理、水利等工程移民安置、扶贫开发移民搬迁、农村危房改造、新农村建设等有机结合起来，促进人口向小城镇聚集。

　　着力提高城市管理水平。推行精细化城市管理，推动城市管理重心进一步向社区下移。深入开展“整脏治乱”，继续规范公交车和出租车经营秩序。突出抓好水城河综合治理，加快污水收集管网建设，完成污水处理厂中水回用工程，加大两岸公园化建设力度，力争水城河面貌有较大改观。

　　（五）大力推进节能减排和生态环境建设，增强可持续发展能力。坚决把住环境评价这道关口，坚决守住保护青山绿水这条底线，走生产发展、生活富裕、生态良好的文明发展之路。

　　继续打好节能减排攻坚战、持久战。大力实施结构性节能减排。加大落后产能淘汰力度，重点整治煤炭洗选企业，依法取缔关闭一批环保不达标、无合法用地手续、达不到产业准入标准的洗选企业。鼓励、支持发展低碳经济和循环经济，推进粉煤灰、煤矸石、矿井水、煤层气等大宗工业废弃物的规模化综合利用，重点推进煤层气综合利用，尽快制定发展规划，促进煤层气的产业化发展。大力实施管理性节能减排。严格执行国家产业政策和行业准入标准，实行环境影响评价、容量总量控制、“三同时”和排污许可证制度，从源头上控制高耗能、高污染、低效益的落后产能进入。充分运用节能减排的各项法律法规和政策措施，加强对重点能耗企业的执法和行政监察，督促企业落实节能减排的主体责任。大力实施工程性节能减排。加大技术改造力度，推广节能减排新技术、新工艺、新设备。加强对燃煤电厂脱硫设施的运行管理，确保完成二氧化硫减排任务。建成市污水处理厂二期工程及平寨、红果、城关、双水、德坞５个县级城镇污水处理厂。在有条件的城镇生活小区规划建设点源污水处理站，促进污水就地收集、就地处理、就地回用。

　　加强生态建设。巩固退耕还林成果，落实国家退耕还林补助延长政策；继续实施天然林保护、珠江防护林等林业生态建设工程，完成１０万亩营造林任务。全面推进石漠化综合治理，完成５１．８８平方公里的年度治理任务。建立健全生态补偿机制，抓好土地、矿产、水资源的保护。加强地质灾害防治，对现有企业造成的地质灾害，按照“谁开发、谁破坏、谁治理”的原则，督促企业进行治理；对历史原因、自然因素造成的无法确定责任主体的地质灾害，由各级政府多渠道筹集资金予以治理；对在村寨下面的采矿项目，必须实行“先搬迁后采矿，不搬迁不采矿”。

　　（六）继续深化重点领域改革，进一步扩大对外开放。着力完善农村综合改革，完成农村集体林权制度改革；在保持现有土地承包关系稳定长久不变的基础上，探索土地承包经营权流转的有效办法，鼓励发展多种形式的适度规模经营。坚持统筹兼顾、分类指导的原则，有序推进市属国有企业改革脱困工作，通过建立“市属国有企业改革专项基金”，逐步解决困难企业职工的生活和社会保险问题；同时，加快县（特区、区）属国有企业改革。深化投融资体制改革，整合国有资产，组建产业投资公司、担保公司和小额贷款公司。大力推进财税体制改革，强化部门预算的刚性约束，加强非税收入及“收支两条线”管理；配合做好增值税转型改革及燃油税改革的相关工作。积极稳妥推进价格改革，适时调整水价、城市生活污水处理费、垃圾处理费；加强对涉及国计民生的物资储备，增强政府对市场物价的平抑能力。按照国家和省的部署，完成市、县政府机构改革。继续深化行政审批制度改革，进一步规范行政审批行为。

　　大力实施“走出去、引进来”的对外开放战略，注重引进实力雄厚的外来战略投资者，借助其资金、先进技术、管理经验和广阔市场，实现共同发展。发挥我市铁路公路的枢纽作用，加强与昭通、曲靖、宜宾、安顺、兴义、毕节等周边城市的交流与合作，把我市建成具有一定辐射范围的物流中心，为融入更高层次的经济圈奠定基础。认真研究发达地区资金流向和产业转移动向，利用我市能矿组合等方面的比较优势，有针对性地开展招商引资。改善投资软环境，努力把我市建成全省审批少、服务好、环境优、效益高的地区，让客商进得来、稳得住、能挣钱。加强对外贸易，不断扩大特色产品出口。加大“凉都”城市品牌打造和对外宣传推介力度，进一步提高对外知名度。

　　（七）进一步抓好生产安全工作，促进安全生产形势持续稳定好转。牢固树立“抓经济建设是政绩，抓安全生产也是政绩”的理念，正确处理安全与生产的关系，切实做到不安全不生产，抓安全促生产。全面贯彻落实“安全第一、预防为主、综合治理”的方针，以“安全生产年”活动为主线，以“三项行动”和“三项建设”为主要内容，严厉打击非法违法生产行为，严格规范企业安全生产行为，严格落实安全生产主体责任和安全监管责任，严格事故责任追究和行政问责，推动安全生产的各项措施落实到生产的各个环节和岗位，努力减少一般事故、控制较大事故、严防重大事故、杜绝特别重大事故。继续以煤矿、道路交通、消防等行业和领域为重点，深化隐患排查治理，扎实打好煤矿整顿关闭和瓦斯治理攻坚战，强化道路交通安全的“五整顿”和“三加强”工作措施。深入开展“质量和安全年”活动，强化食品药品监管，确保群众吃上用上放心的食品和药品。完善落实安全生产的各项规章制度，用制度管人，用制度管事，加快实现安全生产监督管理的常态化、制度化。

　　（八）以保障和改善民生作为保增长的出发点和落脚点，加强社会建设。关注民生、重视民生、保障民生、改善民生是政府的基本职责，我们将把更多的精力用在解决民生问题上。

　　第一，加强社会保障。坚持把扩大就业作为重中之重的任务来抓，抢抓中央扩大内需机遇，积极引导劳动力在基础设施建设等领域就业；大力开拓就业岗位，积极开展针对“４０５０”人员、“零就业”家庭、失地农民、残疾人、返乡农民工等困难群众的就业援助，统筹做好大中专毕业生、复员退伍军人的就业安置，力争全市城镇新增就业１．８万人以上；努力稳定现有就业岗位，特别是在企业改革改制过程中，坚持多兼并、少破产，鼓励优强企业兼并重组濒临破产企业，帮助企业职工实现就业。着力完善社会保障体系，稳步提高城镇职工基本养老、基本医疗、失业、工伤、生育保险的覆盖率和保障水平，逐步扩大城镇居民基本医疗保险覆盖面，积极探索建立农村养老保险制度，启动农民工养老保险；继续做好城乡低保工作，确保动态地实现应保尽保、分类施保。进一步健全社会救助体系，认真落实农村五保供养政策；加大城乡医疗救助力度，缓解困难群众看病难问题；建立健全临时救助制度，努力保障农村贫困家庭、城镇困难家庭、离退休职工、在校贫困学生基本生活水平。扎实抓好保障性安居工程，高质量抓好农村危房改造工作，完成农村茅草屋改造；加快建设廉租住房和经济适用住房，新增１０８００户实物配租廉租住房和１２万平方米的经济适用住房，逐步解决人均住房面积１５平方米以下的城镇低收入家庭住房困难；重视和做好城市拆迁安置工作；加快国有重点煤矿采煤沉陷区综合治理步伐；抓好盘江、水城、六枝三个矿区２．３万户煤矿棚户区改造。

　　第二，加强社会事业。坚持教育优先发展，强化政府对义务教育的保障责任，取消城市义务教育阶段学校借读费，免费为农村义务教育阶段学生提供教科书；进一步巩固提高“两基”成果，确保“两基”顺利通过“国检”；加快发展高中阶段教育和职业教育，鼓励、支持和规范民办教育，重视学前教育，关注边远山区少数民族教育和特殊教育，积极发展远程教育和继续教育，进一步做好六盘水师专“升本”工作；继续实施农村薄弱学校改造和农村中小学校舍改造，加强城市学校基础设施建设，稳步推进市中心城区教育资源整合；加强师资队伍建设，进一步提高教师队伍素质。全面深化医药卫生体制改革，加快完善公共卫生体系，进一步扩大新型农村合作医疗覆盖面，力争参合率达９５％以上；做好地氟病防治工作，确保完成病区家庭的炉灶改良任务；深入开展医院“质量管理年”和创建“平安医院”活动，构建和谐医患关系。统筹解决人口问题，加强流动人口管理，进一步夯实人口计生工作基层基础，稳定和降低生育水平，大力提高出生人口素质，着力遏制出生人口性别比偏高势头。加快文化体育公共服务体系建设，广泛开展群众性文化活动，组织好建国６０周年庆典活动和办好凉都消夏文化节；新建１８０个农家书屋；加强非物质文化遗产保护工作；努力提高广播电视工作水平，推进全市有线电视网络资源整合和数字电视整体平移工作；完成５１７５个２０户以上自然村广播电视“村村通”工程。深入开展全民健身活动，支持发展具有优势和特色的竞技体育。加强国防后备力量建设，搞好优抚安置，巩固和发展“双拥”创建成果。推进老龄、妇女儿童和残疾人事业的发展。不断加强人事人才工作。继续做好科技、民族、宗教、侨务、对台、档案、地方志等工作。

　　第三，加强社会管理。深入开展创建“平安六盘水”工程；大力开展“打黑除恶”、“两抢一盗”等严打整治专项行动；坚持打好禁毒人民战争；加快推进“五五”普法，形成全民自觉学法守法用法的社会氛围；加强和改进信访工作，切实把化解矿群矛盾纠纷、妥善安置失地农民作为维护社会稳定的重头来抓，将工农矛盾有度合理地纳入司法渠道予以解决，努力构建和谐矿区。

　　（九）加强政府自身建设，不断提高政府工作水平和工作能力。应对复杂多变的经济形势，做好今年各项工作，对政府提出了新的考验，需要我们进一步加强政府自身建设。

　　第一，恪尽职守，切实履行政府职能。坚持“市场出效率、政府保公平”，加快转变政府职能，全面履行经济调节、市场监管、社会管理和公共服务职能。更加注重运用经济、法律和必要的行政手段，调整经济结构，转变发展方式；更加注重健全行政执法、行业自律、舆论监督、群众参与相结合的市场监管体系，整顿和规范市场经济秩序；更加注重健全突发事件应急管理机制和社会管理网络，提高政府应对和处置突发公共事件的能力和社会管理的水平；更加注重全方位、全过程地为基层、企业和人民群众搞好服务，扩大政府公共服务的覆盖范围，把更多财政性资金投向社会事业和民生工程，把更多公共资源投向农村、基层和困难群众，加快建设服务型政府。

　　第二，依法行政，实行科学民主决策。按照公正执法、严格执法、文明执法的要求，依靠法律法规规范政府行为，严格按照法定权限和程序行使职权、履行职责。完善群众参与、专家论证和集体决策相结合的决策机制，促进依法决策、科学决策、民主决策。自觉接受人大及其常委会的法律监督、工作监督，严格执行人大及其常委会的决议、决定。积极支持政协履行政治协商、民主监督、参政议政职能，广泛听取各民主党派、工商联和无党派人士的意见和建议，认真办理建议、意见和提案。充分发挥工会、共青团、妇联等人民团体的桥梁纽带作用，接受新闻舆论和人民群众的监督。进一步扩大政务公开，完善新闻发布制度，加强电子政务建设，切实提高工作透明度。大力推行行政执法责任制，严格执行行政首长问责制度，从严整治不作为、慢作为、乱作为的行为。

　　第三，解放思想，不断开拓创新。面对不断发展变化的新形势，我们要加强学习，领会好、贯彻好中央和省的各项决策部署，积极主动适应新形势、新任务、新要求；我们要更加解放思想、更新观念，进一步转变不符合、不适应科学发展观的观念和做法，不为已有的做法所束缚，不为传统模式所局限，不为小富慢进而停滞，以更高起点、更高高度、更大范围来谋划发展；我们要用改革创新的精神状态、工作方法和工作作风推进各项工作，敢于打破常规和思维定式，破除阻碍发展的一切思想障碍和观念痼疾，推进跨越式发展；我们要进一步深化对市情的认识，发挥优势，找准位置，在已取得成绩的基础上，秉承发扬、深化拓展、与时俱进、不断创新，用科学发展观进一步完善和理清发展思路，推动发展上台阶。

　　第四，转变作风，推动工作落实。树立正确的政绩观，坚持“一手抓适应，一手抓提高”，既解决最紧迫、最现实的问题，又多做打基础、增后劲、管长远的工作。强化效率就是生命的意识，加强机关效能建设，大力推行首问负责制、限时办结制、责任追究制，坚决克服慢慢腾腾、拖拖拉拉、对付应付、得过且过的思想观念，努力做到在思路更新上先人一拍、在办法措施创新上先人一筹、在体制机制革新上先人一步。坚持问政于民、问需于民、问计于民，以对人民群众高度负责的精神，俯下身子，深入基层，深入群众，深入实际，着力解决事关群众切身利益的热点难点问题。大力精简会议、文件和应酬，腾出更多的精力想大事、议大事、抓督查、促落实。强化组织纪律观念，着力解决政令不畅、执行不力的问题，确保做到令行禁止。发扬“艰苦创业、开放包容、务实创新、和谐发展”的六盘水精神，始终保持不骄不躁、艰苦奋斗的作风。进一步加强行政监察、审计监督，深入开展廉政建设和反腐败斗争，营造风清气正的政务环境。

　　各位代表！回首２００８年，我们难以忘怀；展望２００９年，我们信心百倍。让我们紧密团结在以胡锦涛同志为总书记的党中央周围，以实现六盘水经济社会又好又快发展为共同责任，齐心协力，同舟共济，奋力拼搏，不断开创我市改革发展稳定的新局面，以优异的成绩迎接新中国成立６０周年！
